# Effects of the Ketogenic diet in overweight divers breathing Enriched Air Nitrox

**DOI:** 10.1038/s41598-018-20933-w

**Published:** 2018-02-08

**Authors:** Gerardo Bosco, Alex Rizzato, Silvia Quartesan, Enrico Camporesi, Devanand Mangar, Matteo Paganini, Lorenzo Cenci, Sandro Malacrida, Simona Mrakic-Sposta, Sara Moretti, Antonio Paoli

**Affiliations:** 1 Environmental physiology & medicine Lab, Department of Biomedical Sciences, University of Padova, Padova, Italy; 2 TEAMHealth Research Institute, TGH, Tampa, Florida USA; 3 Emergency Medicine Residency Program, University of Padova, Padova, Italy; 4 CNR Institute of Bioimaging and Molecular Physiology, Segrate (Milano), Italy

## Abstract

Central Nervous System Oxygen Toxicity (CNS-OT) is one of the most harmful effects of Enriched Air Nitrox (EAN) diving. Protective factors of the Ketogenic Diet (KD) are antioxidant activity, the prevention of mitochondrial damage and anti-inflammatory mechanisms. We aimed to investigate if a short-term KD may reduce oxidative stress and inflammation during an hyperoxic dive. Samples from six overweight divers (mean ± SD, age: 55.2 ± 4.96 years; BMI: 26.7 ± 0.86 kg/m^2^) were obtained a) before and after a dive breathing Enriched Air Nitrox and performing 20-minute mild underwater exercise, b) after a dive (same conditions) performed after 7 days of KD. We measured urinary 8-isoprostane and 8-OH-2-deoxyguanosine and plasmatic IL-1β, IL-6 and TNF-α levels. The KD was successful in causing weight loss (3.20 ± 1.31 Kgs, p < 0.01) and in limiting lipid peroxidation (3.63 ± 1.16 vs. 1.11 ± 0.22; p < 0.01) and inflammatory response (IL-1β = 105.7 ± 25.52 vs. 57.03 ± 16.32, p < 0.05; IL-6 = 28.91 ± 4.351 vs. 14.08 ± 1.74, p < 0.001; TNF-α = 78.01 ± 7.69 vs. 64.68 ± 14.56, p < 0.05). A short-term KD seems to be effective in weight loss, in decreasing inflammation and protective towards lipid peroxidation during hyperoxic diving.

## Introduction

Central Nervous System Oxygen Toxicity (CNS-OT) is one of the most harmful effects of Enriched Air Nitrox (EAN) diving and it is related to oxidative stress and inflammation^1,2^. CNS-OT may cause convulsions similar to epileptic seizures, with sudden loss of consciousness, and other symptoms such as nausea, vomiting, palpitations, visual field constriction, tinnitus and auditory hallucinations. Moreover, signs and symptoms are not preventable taking conventional anticonvulsant drugs, as their mechanisms are based on ion channel regulation and GABA enhancement^3^. Although, the effects of hyperbaric hyperoxic exposure on oxidative stress are well known^4^, minimal data has been collected during diving exposure^5^. During the last few decades there has been a growth of evidence that links overweight to a low-grade inflammatory status^6^. Furthermore, excess of caloric intake leads to an accumulation of NADH inside the mitochondria disrupting the proton gradient and leading to an excessive production of reactive oxygen species (ROS)^7^. Physical exercise leads to the production of ROS and seems to influence the production of pro-inflammatory cytokines^8^. In particular, it has been demonstrated that exercises performed in extreme conditions lead to an imbalance in redox homeostasis, increasing oxidative damage at cellular components such as protein, nucleic acid, lipids^9,10^ and may lead to changes in cellular signaling pathways^11^. Scuba diving is a highly demanding physical activity due to the weight of diving equipment, the increased resistance to movement and the extreme environmental condition^12,13^. It has been reported by Perovic *et al*., that cold temperature, hyperoxia and the physical activity lead to an increased oxidative stress^14^. When antioxidant systems are overwhelmed, the cells are unable to scavenge ROS, and the oxidative damage occurs, inducing pathological conditions^15^. The Ketogenic Diet (KD) is a nutritional approach where carbohydrate is limited to 20–30 grams per day or, in general, less than 5% of total daily calories. When this very low carbohydrate regimen is maintained for more than 4–5 days the body starts to produce the ketone bodies (KBs) - acetoacetate (AcAc), β-hydroxybutyric acid (β-HB) and acetone - by a process called ketogenesis^16,17^. Effectiveness (role) of the KD in reducing the incidence of seizures in epileptic patients is well known since the early 20th century^18^. Recently, several KD therapeutic uses have been validated on weight loss, and reduction of cardiovascular risk factors^19^. Moreover, studies in animal models (*in-vivo* tests), showed the efficacy of the KD in many other neurological/neurodegenerative disorders (i.e. Alzheimer’s, Parkinson’s, Huntington’s disease and spinal cord injuries)^20^. Even though, the antiseizures mechanism of the KD remains unclear, the antiflammatory proprieties of the KD are well recognized in the scientific literature^21,22^. Several studies indicate that ketone bodies metabolism is the major mechanism involved in the beneficial effects given by the KD, including blood lipid profiles^23,24^. Recently, the ketone bodies have been shown as powerful signaling molecules that influence gene expression and inflammation (NLRP3 inflammasome) independent of their effect on metabolism^25,26^.

Whilst β–HB and AcAc tend only to decrease mitochondrial ROS production^27^, other studies suggest the possible role of KD in reducing CNS-OT symptoms. D’Agostino *et al*. demonstrated an association between ketosis and increased latency to seizures in adult male rats exposed to hyperbaric oxygen at 5 ATA^28^. CNS oxygen toxicity can be mitigated (delayed) by Antiepileptic drugs (AEDs)^29^ but Ketogenic diet is an established and effective non-pharmacological treatment for drug-resistant epilepsy^30^. One advantage of the KD is a reduction of potential side effects and impaired physical and cognitive performance associated with AEDs. The first experiment on human subjects undertaken by Valadao *et al*. tested the KD in high partial pressure oxygen diving, obtained good outcomes in terms of tolerance of the KD and no seizures or sign of CNS-OT during immersions^31^. Suggested protective factors of the KD are antioxidant activity, the prevention of mitochondrial damage and the activation of anti-inflammatory mechanisms^31^, however, the cross-talk among all these aspects remain unelucidated.

The aim of our pilot study was to evaluate if a ketogenic state induced by a specific dietary regime, may help divers to balance the negative effects of an excessive oxidative stress damage and inflammatory status during diving activities.

## Results

### Anthropometric, Ketonemia and Glycemia parameters

Six divers (all males) successfully completed the study. After a week of being on the KD, finger-stick testing (through reagent strips) revealed that all divers had attained a moderate ketosis (Table 1). Divers tolerated the diet well and no side effects were registered. All divers experienced a significant weight loss (−3.8%, *p* < 0.01), (Table 2). Ketonemia (mean ± SD: 0.11 ± 0.19) and glycemia (mean ± SD: 87.40 ± 3.7) before the KD was in the normal. Blood glucose levels were normal, with no findings of hypoglycemia (defined as blood glucose lower than 50 mg/dl). Both Ketonemia and Glycemia levels showed no significant differences comparing pre and immediate post-single-dive ketosis (Table 1). There were no apparent aftermaths deriving from implementation of KD. Any neurological or cardiorespiratory effects have been reported after all diving conditions.

**Table 1:** Ketonemia and Glycemia values from finger-stick collection. For both ketonemia (β–HB) and blood glucose, the mean values ± SD (n = 6) measured before (PRE) and after (POST) in-ketosis immersion are shown. In particular, ketonemia values at time T_3_ indicate that all divers achieved a moderate ketosis. Statistics of their differences (ns) is also reported.

SUBJECT	KETONEMIA (mmol/L)		GLYCEMIA (mg/dL)	
	PRE (T3)	POST (T4)	PRE (T3)	POST (T4)
1	0.9	0.8	101	94
2	1.1	1.1	101	103
3	0.8	0.7	98	95
4	0.9	0.9	93	92
5	1.2	1.2	91	95
6	1	0.9	83	82
**MEAN ± SD**	**0**.**98 ± 0**.**15**	**0**.**93 ± 0**.**19**	**94**.**50 ± 6**.**98**	**93**.**50 ± 6**.**77**
**P value**	**ns**		**ns**

**Table 2:** Anthropometric values. For both mass (kg) and BMI (kg/m^2^) the mean values ± SD, (n = 6) of anthropometric values measured before (PRE) and after (POST) Ketogenic Diet and their differences are indicated. Statistical index of the BMI differences (*p* < 0.01) and divers’ height are also expressed.

SUBJECT	Gender	AGE (yrs)	Height (m)	MASS (kg)			BMI (kg/m^2^)		
				PRE (T0)	POST (T3)	Δ	PRE (T0)	POST (T3)	Δ
1	M	60	1.9	96.4	91.4	5	26.7	25.32	1.38
2	M	59	1.71	81	78.4	2.6	27.7	26.81	0.89
3	M	57	1.68	72	68.4	3.6	25.51	24.23	1.28
4	M	48	1.8	85.5	84.4	1.1	26.39	26.05	0.34
5	M	50	1.85	94.9	91.8	3.1	27.73	26.82	0.91
6	M	57	1.7	76.2	72.4	3.8	26.37	25.05	1.32
**MEAN ± SD**	**M**	**55**.**2 ± 4**.**96**	**1**.**77 ± 0**.**09**	**84**.**33 ± 9**.**88**	**81**.**13 ± 9**.**75**	**3**.**20 ± 1**.**31**	**26**.**73 ± 0**.**86**	**25**.**71 ± 1**.**03**	**1**.**02 ± 0**.**39**
**P value**							**<0**,**01**		

### Oxidative damage biomarkers

**Figure 1:**
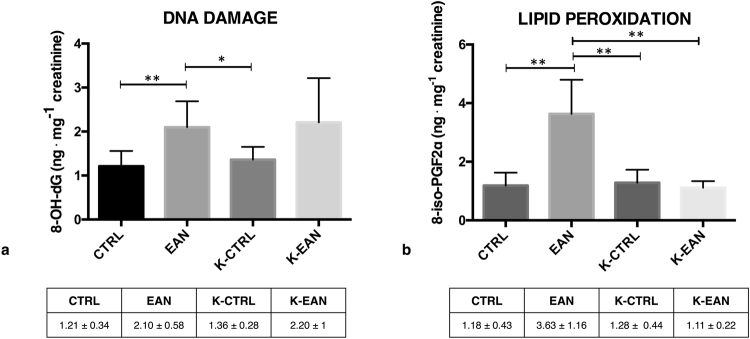
Oxidative damage biomarkers results. (**a**) 8-OH-dG levels at T_0_ (CTRL) and after dive at T_1_ (EAN), after ketogenic diet at T_3_ (K-CTRL) and dive at T_4_ (K-EAN); (**b**) Isoprostane levels at T_0_ (CTRL) and after dive at T_1_ (EAN), after ketogenic diet at T_3_ (K-CTRL) and dive at T_4_ (K-EAN). Vertical bars represent standard deviation. **Significantly different (*p* < 0.01).

Figure 1 shows oxidative damage through 8-OH-dG (DNA damage) and 8-isoPGF2α (lipid peroxidation) tested at each urine collection time. Our results (Fig. 1a) show, a significant increase + 73% (*p* < 0.01) of 8-OH-dG levels during the dive, breathing EAN and compared to the K-CTRL condition (+53%; *p* < 0.05). No significant differences were identified during other comparisons. Similar non-significant differences were identified between CTRL vs. EAN (77%) and K-CTRL vs. K-EAN (67%). In studies measuring lipid peroxidation (Fig. 1b), a significant increase of isoprostane levels during the dive breathing EAN respect to the control condition +208% (*p* < 0.01) and to the ketosis condition +206% (*p* < 0.01) was shown. Instead, a significant decrease −69% (*p* < 0.01) after the in-ketosis dive breathing EAN respect to the only EAN condition was observed. The percentages calculated between CTRL vs. EAN (+244%) and K-CTRL vs. K-EAN (−5%) are significanty different (p < 0.01).

### Inflammatory state biomarkers

**Figure 2:**
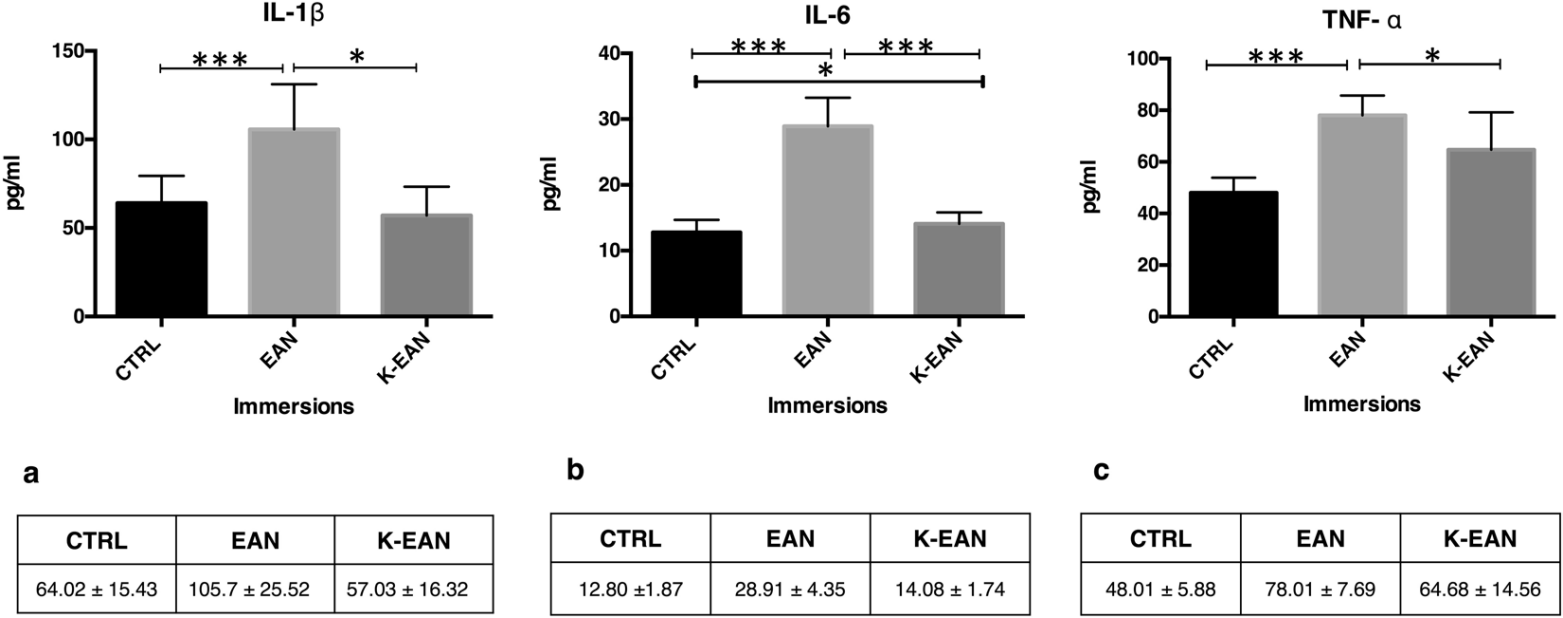
Inflammatory state biomarkers results. (**a**) IL-1β plasmatic levels at T_0_ (CTRL) and after dives at T_1_ (EAN) and T_4_ (K-EAN); (**b**) IL-6 plasmatic level at T_0_ (CTRL) and after dives at T_1_ (EAN) and T_4_ (K-EAN); (**c**) TNFα plasmatic levels at T_0_ (CTRL) and after dives at T_2_ (EAN) and T_4_ (K-EAN). Vertical bars represent standard deviation. *Significantly different (*p* < 0.05), ***significantly different (*p* < 0.001).

Figure 2 shows results of inflammatory state biomarkers (IL-1β, IL-6 and TNF-α) compared at each blood collection time. The basal levels of IL-1β, IL-6 and TNF-α resulted significantly higher compared to normal values usually accepted as reference in literature^32,33^. As reported in Fig. 2a, a significant IL-1β increase after dive-breathing EAN compared to the baseline was measured +65% (*p* < 0.001). Moreover, blood collection following in-ketosis immersion revealed significant values decrease compared to the preceding dive −46% (*p* < 0.05). Furthermore, IL-6 and TNF-α levels showed a significant increase after the dive, breathing EAN with respect to the control condition +126% and +62% (*p* < 0.001, *p* < 0.001) respectively. Besides, values were significantly lower after the in-ketosis immersion in comparison with the first dive respectively for IL-6–51% (*p* < 0.001) and TNF-α-17% (*p* < 0.05).

## Discussion

Recently, an increasing amount of evidence suggests that the KD has beneficial effect on diving mission^31^ and weight loss^34^. There are several mechanisms that may mediate the positive effects of KD including mitochondrial biogenesis and a decrease of inflammation condition.

The excessive protein and lipid oxidation leads to cellular degeneration and functional impairment. The antinflammatory properties of the KD bring ketone bodies to disrupt the inflammasome assembly. In particular, ketones such as β-hydroxybutyrate may regulate inflammation through activities on mitochondrial targets^35^. Additionally, the KD has been shown to decrease proinflammatory cytokine levels after an immune challenge^36^.

Previous studies showed the correlation between cellular redox imbalance and the disease state^6,37,38^. Moreover, aging may be due to a long-term oxidative process, as well as diabetes and atherosclerosis. The effects of scuba diving, on redox homeostasis are still controversial, due to studies conducted under different condition and evaluating a selection of biomarkers of oxidative status^14^.

It has been proposed that physical activity in diving may enhance antioxidant activity^5^, but studies supporting this hypothesis are lacking; little is known on how production of free radicals depends on the diving depth, cold temperature, the duration of exposure to high pressure environment, and in particular the kind of gas breathed. The oxidative damage assessment after EAN exposure highlights that physical exercise and the specific mixture breathed lead to a significant increased oxidative stress^39^ identified in DNA damage and lipid peroxidation levels (see Fig. 1). Moreover, a significant increase in the circulating levels of pro-inflammatory cytokines IL-1β, IL-6 and TNF-α, is observed (see Fig. 2). Previously, we showed that only the KD plus ω-3 reduced the IL-1β and IL-6 levels; while TNF-α decrease during the KD with or without supplementation of ω-3 in overweight subjects without physical effort^40^. To our knowledge, this is the first study that evaluates the effects of a short time KD, to oxidative stress and inflammation, ahead of a recreational or technical diving activity. Free radical oxidation has a prominent role in cardiovascular diseases through their oxidative modification of existing molecules. In particular, isoprostane are markers of lipid peroxidation, and have been clearly linked with cardiovascular, metabolic and neurodegenerative diseases^41^.

Moreover, results showed a significant decrease of 8-isoprostanes at K-EAN time-point with respect to EAN condition and the CTRL condition (Fig. 1); this could be considered a protective effect of KBs on free fatty acids after EAN diving. Inflammatory biomarkers, IL-1β, IL-6 and TNF-α decreased in the comparison between K-EAN vs EAN immersions (Fig. 2) suggesting that a short term KD could play a role on inflammatory state in scuba diving. Multiple hypotheses and several mechanisms have been proposed to elucidate the anti-inflammatory effects of a KD^19,40,42^.

Conversely, different responses were found on 8-OH-dG in EAN and K-EAN dives. DNA damage increases in EAN and in K-EAN with respect to the CTRL (Fig. 1). The increase of 8-OH-dG measured at T_1_ showed an increased DNA oxidation induced by hyperoxia. Accordingly, Gröger and colleagues showed that prolonged normobaric and hyperbaric pure O_2_ breathing, caused oxidative DNA damage and lipid peroxidation^43^.

This pilot study highlights that a short term KD does not significantly affect (CTRL vs. K-CTRL) the oxidative state of both DNA and lipds. However, we observed a significant lowering lipid peroxidation in EAN diving induced by ketosis intervention. Overall, the KD could be considered a safe diet regimen that contributes to achieve healthy conditions, reducing body weight and regularizing BMI. We observed a decreased inflammatory status in the EAN diving condition, however, we can only speculate that a short-term KD alone could reduce the levels of inflammatory markers.

## Conclusions

A well-controlled and a short duration KD could be used in recreational or technical diving without any clear contraindications. Indeed, none of the divers had any ill effects during KD and EAN diving in ketosis. However, further studies are needed on a larger sample size investigating also inflammatory markers after KD, in order to confirm these preliminary results. Moreover, a comparison between KD and a hypocaloric non ketogenic diet would be useful to differentiate if observed results could be attributable simply to a caloric restriction or to a specific diet regimen.

## Methods

### Experimental design

The experimental protocol received the approval by the local Human Ethical Commitee (n° HEC-DSB 06/16) of the Department of Biomdical Science of University of Padova and adhered to the principles of the Helsinki Declaration. Written informed consents were obtained from the divers before enrollment in the study. The study registration number in the ClinicalTrials.gov register is NCT03114176 (04/10/2017).

**Figure 3:**
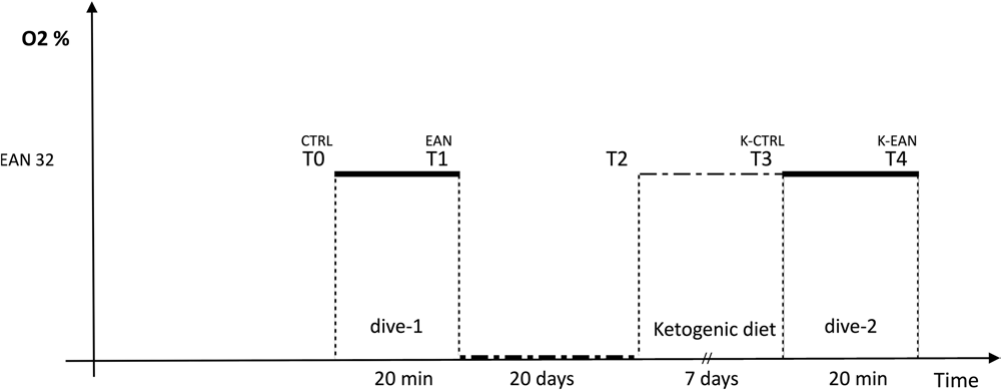
Experimental Design. T_0_: blood and urine sampling and anthropometric measures at Base Line (control); T_1_: urine and blood sampling after immersion breathing Nitrox; T_2_: beginning of Ketogenic Diet; T_3_: urine sampling, ketone bodies and blood glucose sampling and anthropometric measures when the diet ended; T_4_: urine, blood, ketone bodies and blood glucose sampling after a dive breathing Nitrox at final time point of the study.

This reaserch was a pilot study. It is a prospective interventional controlled experimental design, with repeated measures conducted under three different experimental conditions. Fourteen days before the beginning of the testing sessions, a familiarization meeting was organized to ensure that all the divers knew the protocol and could complete the scheduled program. In particular, the divers were instructed about the experimental protocol and were provided of a diet plan to control dietary intake during the planned week. Rules for usage and principles of KD were explained by an expert dietician. During the experimental period, divers were informed to refrain from heavy physical activity. Throughout each dive, divers performed a 20-minute long mild exercises on an underwater bike (OKEO, Genoa, Italy). The depth of dive was set at 15 meters, where the divers performed an activity guided by Borg CR-10 scale at intensity level 3 (25 rpm)^44^. The ascent rate was set at 10 m/min, with a decompression stop at 5 meters for 3 min, according to the US Navy Manual Diving Table. 48 h prior to the immersions, none of the divers consumed medications or dived or flew; they refrained form physical activity during the two weeks preceding the trials. In the first part of the experiment, all divers performed a dive breathing Enriched Air Nitrox (EAN, 32% FiO_2_). Baseline clinical measurements were collected before and after this first dive (T_0_ and T_1_, Fig. 3) in order to have a reference [CTRL] and to measure physiological modifications due to immersion using EAN. After twenty days of no diving activity, divers were engaged in a KD for seven days (T_2_). At the end of this period [K-CTRL], urine samples were collected. Then, divers performed a single immersion breathing EAN. The measures were performed after this single dive [K-EAN] at time T_4_ (Fig. 3). The experimental setting for the trials was the world’s deepest pool “Y-40 THE DEEP JOY” with a water temperature of 31–32 °C located in Montegrotto Terme (Padua, Italy).

### Subjects

Initially, twelve diving instructors were enrolled and screened medically. Inclusion criteria were: to be in an overweight state (BMI ≥ 25 kg/m^2^) and no history of orthopedic, cardiovascular, renal or metabolic disorders. Six divers were excluded from the study because they didn’t match the inclusion criteria. In particular, four were obese, one had recent cardiovascular disorders and one was not available in completing the amount of work required in the study. Finally, six male divers (mean ± SD, age: 55.2 ± 4.96 years; body weight: 84.3 ± 9.88 kg; height: 1.77 ± 0.09 m; BMI: 26.7 ± 0.86 kg/m^2^; 50 dives/year in the last two years) were selected for the study. All of the divers gave their written informed consent.

### Testing session and samples collection

Every testing session was characterized by a dive in which subjetcs performed an underwater exercise of mild intensity. Moreover, blood and urine samples were collected from each diver in a mobile laboratory respectively in T_0_, T_1_, T_4_ and T_0_, T_1_, T_3_, T_4_ (Fig. 3). In detail, approximately 10 mL of blood was drawn from an antecubital vein and collected in heparinized (10 mL) treated vacutainer tubes (Becton Dickinson and Company, UK). Plasma was separated by centrifugation (5702 R, Eppendorf, Germany) at 3000 g for 5 min at 4 °C. Samples of plasma were then stored at −80 °C. Also, urine samples collected were stored at −80 °C until analyses were performed. Analysis on biological samples were performed within two weeks from collection.

A competitive immunoassay was used for the determination of 8-isoprostane (8-iso-PGF2α) concentration, a marker of lipid peroxidation, in urine (Cayman Chemical, USA). Urine was purified using solid phase extraction cartridges. The purification and the subsequent EIA assay were performed following the manufacturer’s recommendations. The EIA employs 8-iso-PGF2α tracer and 8-iso-PGF2α antiserum. The sample 8-iso-PGF2α concentration was determined using a standard curve. Samples and standards were read at a wavelength of 412 nm. 8-OH-2-deoxyguanosine (8-OH-dG) has been established as a marker of oxidative DNA damage. This compound was quantified in excreted urine. A commercially available enzyme immunoassay EIA kit (Cayman Chemical, USA) for the measurement of 8-OH-dG was utilized. The EIA employs an anti-mouse IgG-coated plate and a tracer consisting of an 8-OH-dG-enzyme conjugate. The sample 8-OH-dG concentration was determined using an 8-OH-dG standard curve. Samples and standards were read at a wavelength of 412 nm. Urinary concentrations of 8-iso-PGF2α and 8-OH-dG, as any urinary marker, vary considerably; therefore, the urinary parameters are usually standardized basing on the amount of creatinine excreted in the urine when the collection of the 24 h urine is not possible. Indeed, in the absence of renal disease, the excretion rate of creatinine in an individual is relatively constant. Thus, urinary creatinine levels may be used as an index of standardization. Creatinine assay kit (Cayman Chemical, USA) was used to measure creatinine levels in urine samples. Creatinine concentration was determined using a creatinine standard curve.

Interleukin-1 β (IL-1β), Interleukin 6 (IL-6), and tumor necrosis factor alpha (TNF-α) plasmatic levels were determined by ultrasensitive ELISA kits (R&D Systems, Minneapolis, MN, USA), according to the manufacturer’s instruction. Briefly, a monoclonal antibody specific for the inflammatory marker of interest (IL-1 β, IL-6 or TNF-α) was pre-coated onto a micro-plate (one plate for each marker). Standards and samples (~200 μL) were pipetted into the wells and the immobilized antibody bound any antigen of interest (IL-1 β, IL-6 or TNF-α) present. Following the washing procedure, an enzyme-linked, specific for the investigated antigen, polyclonal antibody was added to the wells. After subsequent washing, a substrate solution was added to the wells and color developed in proportion to the amount of cytokine bound at the initial step. The signal was then spectrophotometrically measured at a wavelength of 450 nm.

### Ketogenic Diet

Informations on body weight, height and BMI were acquired before starting the trials (T_0_ in Fig. 3). After the first session of tests (T_2_) divers started the KD, lasting one week, following the menu provided by a qualified dietician during an individual visit (Table 3).

**Table 3:** Ketogenic Diet plan. Table summarizes the diet schedule for divers. For each meal of the day recommended food are precisely reported. Additional special food protein and fibers based produced by Tisanoreica (Gianluca Mech SpA, Italy) are included in the program. Tisanoreica Alimentary Portion (TAP) is the unit of measurement used to define the amount of amino acids contained in each food preparation. Each Tisanoreica Alimentary Portion contains about 19 grams of protein (obtained from milk, eggs, peas, soy) equivalent to 1 TAP.

Meal	Foods
*Breakfast*	1 or 2 cups of coffee or tea without sugar (stevia and aspartame are allowed) + 1 Tisanoreica Alimentary Portion (TAP)
*Snack* (*mid-morning or afternoon*)	1 or 2 cups of coffee or tea without sugar or sweetener + 1 Tisanoreica Alimentary Portion (TAP)
*Lunch* (*lunch and dinner can be reverse*)	• 2 oz of Tisanoreica Tisanopasta Fusilli, Penne or Rice (2 TAP);
	• raw or cooked vegetables chosen from asparagus, beetroot, broccoli, artichokes, thistle, chicory, palm-hearts, cauliflower, cabbage, Brussels sprouts, cucumbers, sauerkraut, sorrel, turnips, water-cress, fennel, cultivated mushrooms, soy sprouts (fresh), endive, salad (Belgian, lettuce, escarole, curly endive, valerian, etc.), eggplant, leeks, radicchio (green), radishes, rocket, celery, spinach, Savoy cabbage, truffles, zucchini.
*Dinner* (*lunch and dinner can be reverse*)	• Choose an alternative: - 0.22 lb of meat with no visible fat chosen from: chicken, turkey, rabbit, veal, beef, foal, frog, quail, pheasant, duck, lamb, Guinea fowl; - 0.33 lb of clean fish at your choice from: cod, tuna (fresh, in oil or in brine), mackerel, carp, sole, pike, grouper, bass, scorpion fish, squid, ink-fish, ray, dentex, salmon, gilthead, lobster, mullet, turbot, smooth hound, octopus, anchovy, clams, crab, shrimps, mussels, oysters; - 0.13 lb of cold meats (air-cured beef, carpaccio, defatted raw ham); - 2 eggs;
	• Cooked or fresh vegetables at pleasure (see lunch).
**DRESSINGS**	
Extra-virgin olive oil (2 spoonfuls daily), lemon juice (2 spoonfuls daily).	

Divers were instructed to consume at every meal the predetermined pack of food and liquids . A dietitian was enrolled in order to control their food intake and to explain and deliver all the food portions to the divers. More than this, a researcher contacted the divers regularly by phone during the selected week (twice a day) and the dietitian (once a day). Overall, the diet consisted of a very low carbohydrate ketogenic diet (for 7 days) with the use of some phytoextracts as previously described^40,45,46^. We added the phytoextracts in order to have the same quality and quantity of liquid for all divers.

The consumed diet was primarily made of beef and veal, poultry, fish, raw and cooked green vegetables without restrictions, cold cuts (dried beef, carpaccio and cured ham), eggs and seasoned cheese (e.g., parmesan). The allowed drinks were infused tea, moka coffee and herbal extracts. The foods and drinks that divers had to avoid included alcohol, bread, pasta, rice, milk, yogurt, soluble tea, and barley coffee. In addition, to facilitate adherence to the nutritional regime, a variety of special meals based on protein and fibers were given to each subject, produced by Tisanoreica (Gianluca Mech SpA, Asigliano Veneto, VI, Italy). KD characteristics are protein intake 1.3 g/kg/day, to maintain skeletal muscle mass; carbohydrates intake less than 40 g/day and 50–60% of total kcal to induce ketosis (Table 3)^47,48^. In accordance with a hypocaloric profile, total energy intake was calculated with 200 kcal more than basal metabolic rate estimated with Harris-Benedict formula^49^. Body weight was measured also at time T_3_, when KD ended (Fig. 3). The ketosis state of each subject was ascertained by testing Beta-hydroxybutyrate (β-HB) through a finger-stick (FreeStyle, Optium β-Ketone, Abbott, England) (T_3_) the assay was repeated after the immersion, at time T_4_. Furthermore, glucose levels were assessed (T_3_ and T_4_), through a finger-stick (FreeStyle, Optium Neo, Abbott, England). We used the values suggested by the manufacturer (FreeStyle, Optium β-Ketone, Abbott, England) as reference for nutritional ketosis (low: 0.5–0.9 mmol/L; mid: 1.9–2.9 mmol/L; high: 3.5–5.5 mmol/L).

### Statistical Analysis

All data are expressed as mean ± Standard Deviation (SD) and compared with the statistical GraphPad Prism software (GraphPad Prism 6, Graphpad Software Inc., San Diego, CA). After a normality test (Kolmogorov-Smirnov), data were analyzed with a parametric test for multiple comparisons (one-way ANOVA) to determine differences between dives and the control condition both for inflammatory and oxidative stress biomarkers. Two-tailed student’s t-test was also used to compare values of BMI, Glycemia and Ketonemia before and after the in-ketosis dive. *P* < 0.05 was considered significant.
